# Perianal Tailgut Cyst: An Unusual Presentation

**DOI:** 10.7759/cureus.27512

**Published:** 2022-07-31

**Authors:** Rabia Arshad, Noor Khalid, Mubashir Rafique, Ruqia Mushtaq, Fakhar Munir Sial

**Affiliations:** 1 General Surgery, Benazir Bhutto Hospital, Rawalpindi, PAK

**Keywords:** perianal cyst, pedunculated mass, perianal lesion, tailgut cyst, surgical case reports

## Abstract

A tailgut cyst (TGC) is a rare congenital lesion that occurs due to failure of involution of the distal hindgut, leading to the development of a mucus-secreting cyst. The clinical presentation is nonspecific, and often the diagnosis can be missed. We present the case of a 20-year-old female with a TGC in the perianal region. Surgical excision of the cyst was performed, followed by an uneventful recovery. The young age of our patient and the anatomical location of the TGC make our case a rare entity, highlighting the need for practicing surgeons to keep TGC as a differential in mind while examining masses in the perianal region.

## Introduction

Tailgut cysts (TGCs) are rare congenital lesions that occur due to failure of involution of the distal part of the hindgut, which normally regresses in the embryonic period. This leads to the development of mucus-secreting cysts [1]. These cysts are often thin-walled and multilocular and may measure up to several centimeters in diameter. TGCs are usually located in the retrorectal or presacral space as asymptomatic or mildly symptomatic cystic masses [1]. These lesions may rarely be present in the perianal space [2]. The epidemiological data available for TGCs demonstrate a 5:1 predominance of these lesions in females [2], with some centers reporting this ratio to be as high as 7:1 [3]. TGCs are rare, and an incidence ratio of 1/40000 hospital admissions has been described by Mayo Clinic experience data spanning 20 years [4]. The mainstay of treatment is surgery. While diagnosis is usually made postoperatively via histopathology, the multilocular nature of these lesions and thick brownish fluid content can be used as identifying features for TGCs [2].

## Case presentation

**Figure 1:**
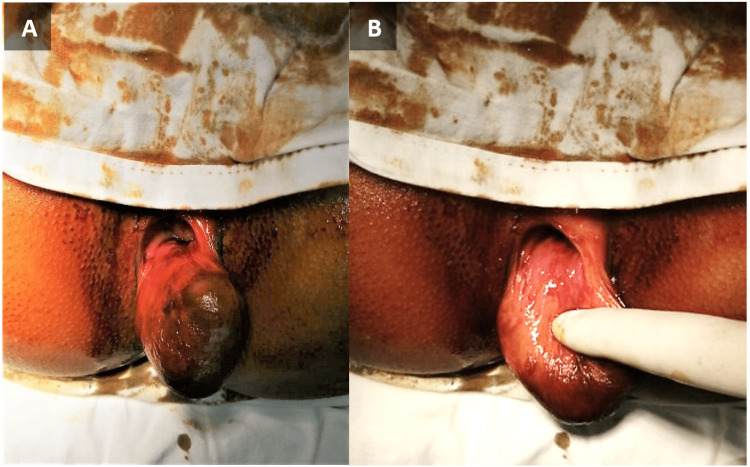
Tailgut cyst. Images 1A and 1B show the patient in lithotomy position with the tailgut cyst protruding out of the anal canal. It had a soft consistency and a smooth surface covered with skin.

A 20-year-old female patient presented to the outpatient department of Benazir Bhutto Hospital, Rawalpindi, with the complaint of something coming out of her anus since birth, which had increased in size over the past year. It was associated with mild, dull pain in the perianal region. The pain did not exacerbate on defecation. There was no associated history of fever, constipation, per-rectal bleeding, or any urinary complaints. Her past medical and surgical history and systemic inquiry were unremarkable. There was no family history of cancer, and the patient’s drug history was insignificant. On examination, 6 x 4 cm irreducible swelling was seen arising from the anal verge with its pedicle at the 6 o’clock position. The margins of the mass were regular with a smooth surface. The overlying skin was not attached to the swelling. There was no active bleed or discharge (Figure 1).

**Figure 2:**
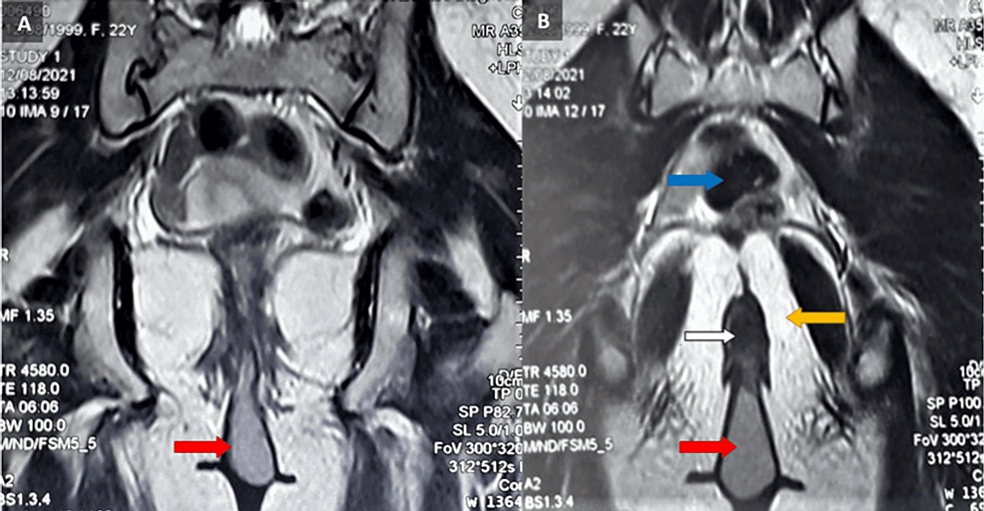
Axial MRI scan of the tailgut cyst. This MRI scan shows a hyperintense lobulated soft tissue lesion (red arrow) in the region of the anal canal (white arrow). It arises from the external anal sphincter on the left side at the 4 o'clock position. It bulges into the anal canal and laterally has a mild mass effect on the left ischiorectal fossa (yellow arrow). Also shown for reference is the sigmoid colon (blue arrow).

The MRI of the pelvis revealed a 5.9 x 5.5 x 3.1 cm circular cystic lesion arising just posterior to the external anal sphincter on the left side with an external mild mass effect on the left ischiorectal fossa and an intact interface. Inferiorly, it protruded out of the anal canal. The rest of the pelvic and abdominal vessels and viscera were normal on MRI. The differential diagnosis of this pedunculated anal swelling included anal polyp or anal lipoma (Figure 2).

**Figure 3:**
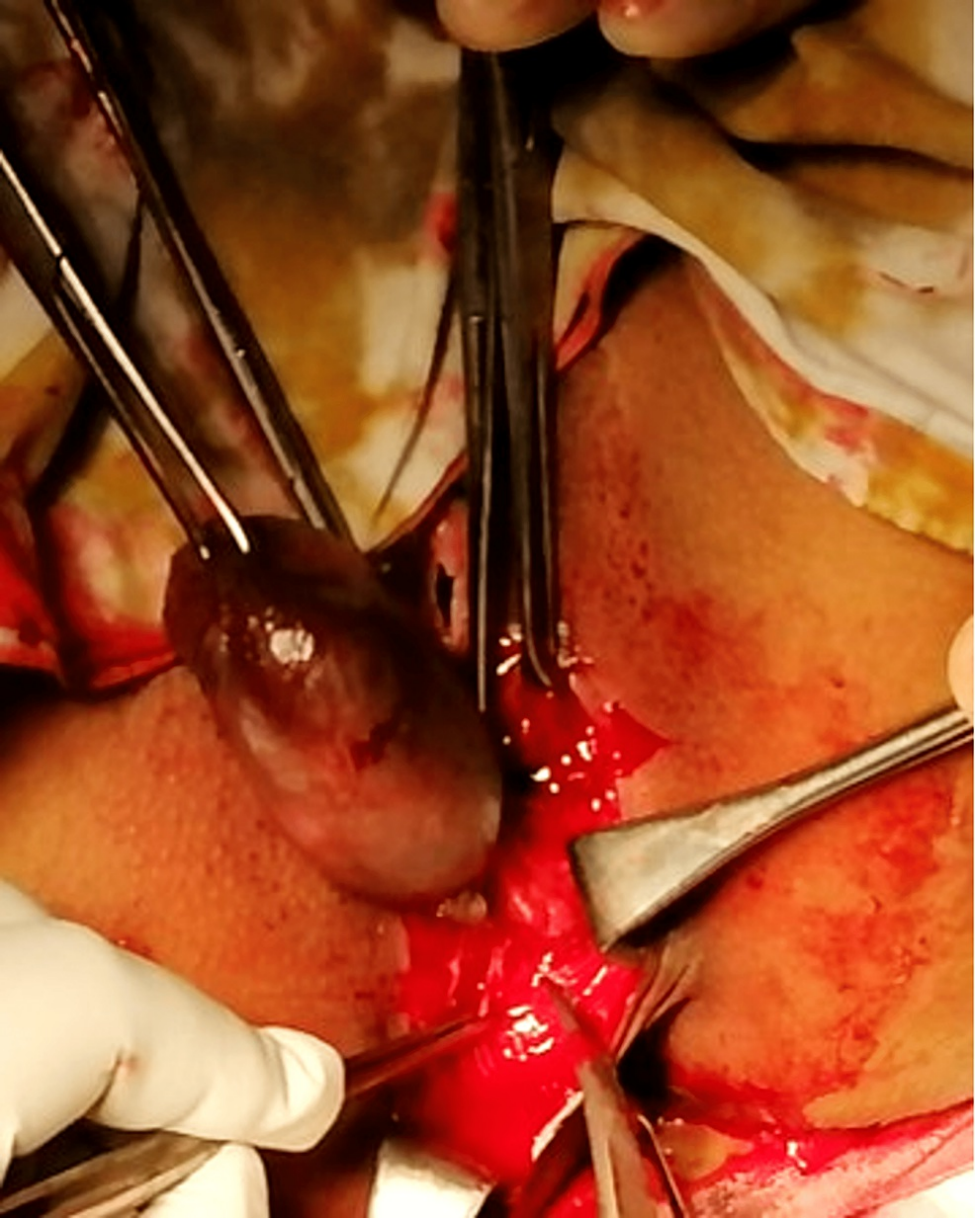
Surgical excision of the tailgut cyst. The figure shows the cyst being dissected out from the surrounding tissues, still attached to the anal verge at its pedicle.

Proctoscopy and sigmoidoscopy done during the preoperative planning demonstrated that the lesion lay externally around the anal canal, with no involvement of deeper structures. Therefore, examination under anesthesia (EUA) and excision was planned. Perioperatively, a broad-based, fluid-filled, pedunculated swelling was seen arising from the anal verge, 2 cm below the dentate line. Using a circumferential incision, skin and subcutaneous tissues were incised, and the pedicle was separated. After the lesion was excised, the structures were closed in reverse order (Figure 3).

**Figure 4:**
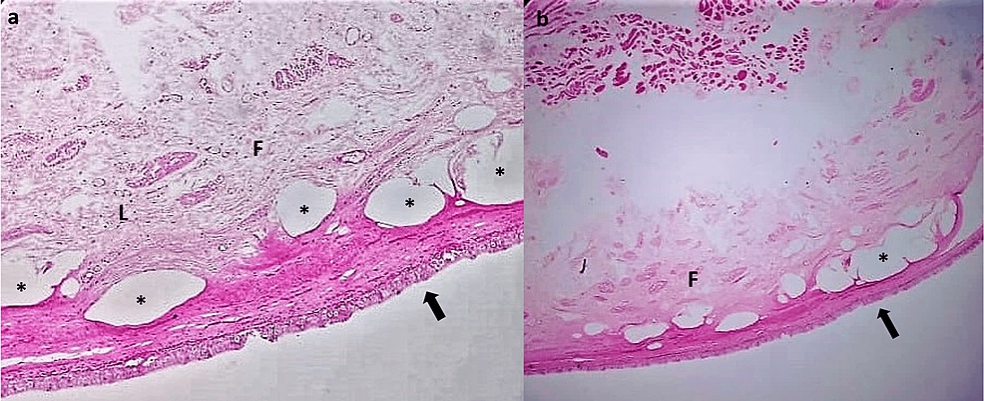
Histopathological sections of the excised specimen. The specimen is lined by stratified squamous epithelium (black arrows) with areas of transitional and pseudostratified ciliated columnar epithelium in between. Both Figures 4A (right) and 4B (left) demonstrate cystic spaces (*) surrounded by fibromuscular stroma (F). Interspersed between the stroma, a variety of lymphocytes can be seen (L).

On dividing the specimen into two halves, the lesion showed a large cyst measuring 4.5 cm in diameter and a smaller cyst measuring 2 cm in diameter; both filled with brown-colored amorphous material. The dissected specimen was labeled accordingly and sent for histopathology. Fluid for culture and sensitivity was also sent. The patient was discharged the next day on analgesia. The fluid culture report showed no growth of organisms. The histopathology sections revealed cystic lesions lined by stratified squamous keratinized epithelium with transitional-to-pseudostratified ciliated columnar epithelium. It was surrounded by focal areas of fibromuscular stroma showing infiltration by foamy histocytes, lymphocytes, and plasma cells. No evidence of malignancy was seen (Figure 4). The findings of this benign cystic lesion were consistent with the TGC.

Postoperative recovery was uneventful, with the patient reporting return to routine activities in a few days. A month later, on her follow-up visit, she had no active complaints and was satisfied with her treatment's medical and cosmetic outcomes.

## Discussion

TGCs are rare congenital lesions that occur due to failure to involution in the most distal part of the hindgut. Embryologically, the part of the hindgut that extends into the tail of the embryo regresses typically by the eighth week of gestation. Failure of this process results in developing a mucus-secreting TGC [1].

TGCs are more common in females and can present at any age, but they have been more commonly described in the fifth decade of life [5]. The clinical presentation is nonspecific, with a large proportion of patients being asymptomatic. Of those symptomatic, the most common complaints are lower back pain, dysuria, increased urinary frequency, painful defecation, constipation, or infection [6]. Long-term risk for malignancy has also been described in the literature, with the rate of malignant transformation reported to be greater than 25% [7].

Grossly, TGCs can be recognized on digital rectal examination as a thin-walled, multilocular cystic lesion protruding from the anus (similar to our patient), or they may be detected incidentally during imaging. They are often large, measuring up to several centimeters in diameter [8]. Microscopically, they are characterized by a mucoid-filled cystic mass lined by a variety of epithelia, including squamous, columnar, and transitional [9].

Various imaging modalities can detect TGCs, including USG and CT. However, MRI remains the gold standard imaging technique for detecting and guiding surgical intervention [5]. On MRI, a TGC is usually multilocular with internal septa. TGCs demonstrate a high-intensity signal on T2-weighted images and a low-intensity signal on T1-weighted images. However, it may appear hyper-dense on T1-weighted images if mucinous material, hemorrhage, or high protein content is present [1,10]. In addition, malignant transformation of TGCs shows irregular wall thickening and polypoid masses on MRI imaging [11].

There are no established guidelines for the management of TGCs. However, complete surgical removal has been recommended for these lesions due to the risk of infection and malignancy. The most common surgical approach used worldwide is open surgical excision using the posterior approach [5]. Postoperatively, histopathological analysis is then used for definitive diagnosis. A preoperative biopsy is not recommended for TGCs [12].

There is extremely limited data regarding the post-excision follow-up of these patients. Some case series have highlighted the risk of recurrence after surgical removal. However, such recurrence has been attributed to incomplete excision, and the actual recurrence rates are low [2,4]. Due to the possible risk, regular follow-up visits are nevertheless the general norm, and a combination of patient history and clinical examination can be used to assess recurrence.

There is a broad list of differentials for TGCs, and more often than not, these lesions are misdiagnosed. The possible list of differentials includes dermoid and epidermoid cyst, rectal duplication cyst, teratoma, chordoma, meningocele, and lipoma, to name a few [1,5,9]. In addition, in case of a bulging perianal mass and the presence of infection, TGCs can be misdiagnosed as hemorrhoids and pilonidal sinus, respectively [13].

Our patient was initially misdiagnosed as having a perianal pedunculated lipoma, with the subsequent differential being external hemorrhoids. Looking at it in retrospect, the diagnosis should have been made preoperatively based on the multilocular cystic nature of the lesion and intraoperatively by the thick brownish fluid drained from the specimen. Grossi U et al. described these findings as being pathognomonic for perianal cysts, with all cases of perianal TGCs reported till date presenting similarly [2].

Our findings are consistent with the published literature with respect to epidemiology, diagnostic difficulty, and management approach. However, there are certain unique aspects to our case. First, our patient had a TGC situated in the perianal region, with its origin 2 cm beneath the dentate line. Most of the TGCs reported in the literature are located in the retrorectal or presacral space [6], and to date, only 10 cases of perianal TGCs have been reported [2]. Furthermore, most of these perianal TGCs have been reported in elderly patients, whereas our patient was a 20-year-old female, which makes our case a unique addition to the pre-existing literature.

## Conclusions

Due to the fact that perianal TGCs are an extremely rare entity, they are often not included in the list of differential diagnoses for perianal swellings. TGCs are often asymptomatic; however, the late diagnosis can be harmful due to the risk of malignant transformation in long-standing TGCs. Therefore, TGCs should always be considered for multilocular cystic swellings in the presacral and perianal regions in female patients, especially if they contain thick, brownish fluid on aspiration. The preferred management is complete surgical excision to avoid recurrence and postoperative histopathology to confirm the diagnosis and rule out neoplasia.
